# Use of Diuretics is not associated with mortality in patients admitted to the emergency department: results from a cross-sectional study

**DOI:** 10.1186/s12952-016-0044-1

**Published:** 2016-02-01

**Authors:** Dominik G. Haider, Gregor Lindner, Michael Wolzt, Alexander Benedikt Leichtle, Georg-Martin Fiedler, Thomas C. Sauter, Valentin Fuhrmann, Aristomenis K. Exadaktylos

**Affiliations:** Department of Emergency Medicine, Inselspital, University Hospital Bern, Freiburgstrasse, Bern, 3010 Switzerland; Department of Clinical Pharmacology, Medical Universtiy of Vienna, Spitalgasse 23, 1090 Vienna, Austria; Center of Laboratory Medicine, Inselspital, University Hospital Bern, Bern, Switzerland; Department of Intensive Care Medicine, University Hospital Hamburg-Eppendorf, Martinistraße 52, 20246 Hamburg, Germany

**Keywords:** Diuretics, Mortality, Critically ill, Emergency department

## Abstract

**Background:**

Patients with diuretic therapy are at risk for drug-induced adverse reactions. It is unknown if presence of diuretic therapy at hospital emergency room admission is associated with mortality.

**Methods:**

In this cross sectional analysis, all emergency room patients 2010 and 2011 at the Inselspital Bern, Switzerland were included. A multivariable logistic regression model was performed to assess the association between pre-existing diuretic medication and 28 day mortality.

**Results:**

Twenty-two thousand two hundred thirty-nine subjects were included in the analysis. A total of 8.5 %, 2.5 %, and 0.4 % of patients used one, two, or three or more diuretics. In univariate analysis spironolactone, torasemide and chlortalidone use were associated with 28 day mortality (all *p* < 0.05). In a multivariate cox regression model no association with mortality was detectable (*p* > 0.05). No difference existed between patients with or without diuretic therapy (*P* > 0.05). Age and creatinine were independent risk factors for mortaliy (both *p* < 0.05).

**Conclusion:**

Use of diuretics is not associated with mortality in an unselected cohort of patients presenting in an emergency room.

## Background

Diuretics are an established pharmacotherapy for diseases such as hypertension, symptomatic heart failure or the nephrotic syndrome. Adverse effects include electrolyte disorders and volume depletion [1]. In an ageing population, the number of patients treated with diuretics is increasing, and is paralleled by side effects [2, 3].

Studies investigating diuretic therapy-induced electrolyte disorders have mainly focused on the effects of thiazide diuretics. In a randomized controlled trial in hypertensive patients, serum potassium levels were significantly lower in patients receiving thiazide diuretics [4]. However, only patients who did not receive potassium supplements developed marked hypokalemia with potassium levels below 3.0 mmol/L [4]. A more recent study on hypertensive patients found an incidence of 30 % for hyponatremia in patients treated with thiazide diuretics [5], which was not associated with an increased risk of hospitalization or death.

Use of diuretic therapy is associated with acute kidney injury or mortality in postsurgical patients [6–8]. While the association of pre-existing diuretic therapy with electrolyte imbalances at emergency room admission has been described previously [9], the relationship with mortality is not known in this group of patients. The present retrospective analysis has therefore studied whether pre-existing use of diuretics at hospital admission serves as an indicator of poor clinical outcome in this population.

## Materials, methods and patients

All patients admitted to the ER of the Inselspital, University Hospital Bern, between 1 January 2009 and 31 December 2010 were included in this cross-sectional analysis. During the study period 22239 patients were enrolled into the study. In the case of multiple admissions, only the first admission to the ER was considered for the analysis. Patient data was anonymized for analysis and de-identified prior to analysis.

For these 22239 patients, data on age, sex, admission type (medical or surgical), pre-existing diuretic medication, country of residence, hospital admission, length of hospital stay, outcome and final diagnosis as classified by the *International Classification of Diseases*, 10th revision (ICD-10) were collected.

Daily dose of the following diuretic medicines was calculated: hydrochlorothiazide, chlorthalidone, butizide, amiloride, spironolactone, eplerenone, furosemide, torasemide, indapamide, metolazone and acetazolamide.

A waiver for informed consent was provided for retrospective analysis of pseudononymized data. The study protocol was approved by the Ethics Committee of the Canton of Bern, Switzerland.

### Statistical analysis

Data are presented as means ± standard deviation (SD), medians or proportions, as appropriate. Between-group comparisons of continuous variables were performed using the Mann–Whitney *U* test.

Pearson’s Chi-Square Test was used for the identification of associations with survival.

Multivariable logistic regression analysis was used to explore the association of the various predictors with the presence of electrolyte disorders and with hospitalization. Predefined covariates were added to the logistic regression models. Cox regression was used to test associations of the diuretics with the survival time adjusted for predefined covariates.

A two-sided *P* value of <0.05 was considered statistically significant for all analyses. The statistical analysis was performed using SPSS (SPSS for Windows V.17.0, Chicago, IL, USA).

## Results

Data from 22239 patients were included in the analysis. The mean age at presentation was 52 years (SD 20) and 57 % were men. 76 % of patients were Swiss residents. Of these 1939 (8.7 %) patients were admitted to the intensive care unit. Reasons for admission were coded via ICD classification and was available in 11898 patients. In 702 patients cancer, in 2884 patients intoxication, in 146 patients endocrinologic, in 1053 patients gastroenterologic, in 522 patients infectious, in 2831 patients cardiovascular, in 650 patients pulmonary, in 546 patients musculo-sceletal, in 517 patients nephrologic/urologic, in 1119 patients neurologic/psychiatric, in 928 patients other (ear, eye, dermatologic) diseases were the reason for admission.

**Fig. 1:**
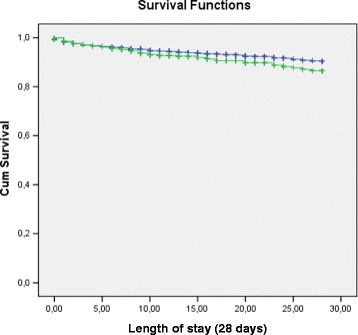
Kaplan**-**Meier curve for mortality in patients with diuretics (*green line*) versus patients without diuretics (*blue line*) mortality within 28 days (*p* = 0.10)

Overall 587 (2.8 %) patients died within 28 days of admission. Diuretic therapy is listed in Table 1. When compared with patients without diuretic therapy, patients with diuretic therapy had a higher mortality (Mann–Whitney-U, *p* < 0.001). However, there was no significant difference in mortality between patients with or without diuretic therapy in overall survival (*p* = 0.088) and in mortality within 28 days in Kaplan Meier analysis (Fig. 1).

**Table 1:** Subject characteristics for diuretic therapy. Data shown as absolute numbers and %

	*n* (%)
Diuretic therapy	2401 (11.3)
Hydrochlorothiazid	936 (4.4)
Chlorthalidon	99 (0.5)
Butizid	11 (0.1)
Amilorid	93 (0.4)
Spironolacton	384 (1.8)
Furosemid	212 (1.0)
Torasemid	1143 (5.4)
Indapamid	52 (0.2)
Metolazon	115 (0.5)
Eplerenon	13 (0.1)
Diamox	20 (0.1)

In univariate analysis ethnicity, pre-existing diuretic therapy, the amount of diuretics, and the medicines chlorthalidon, spironolactone and torasemide were associated with mortality (Table 2, all *p* < 0.05). With exception of furosemide dosage (*p* = 0.02) comparable results were obtained when dosages of the respective diuretics were tested for associations with survival (data not shown). Admission to an intensive care unit (ICU) was associated with mortality (*p* < 0.001).

**Table 2:** Associations of parameters with mortality in univariate analysis, Chi-Square Test for binary and Mann–Whitney *U* test for continuous variables (*p < 0.05)

	*p*-value
Sex	0.074
Ethnicity	0.041*
Diuretic therapy	<0.01*
Amount of diuretics	<0.01*
Hydrochlorothiazid	0.834
Chlorthalidon	0.046*
Butizid	0.527
Amilorid	0.126
Spironolacton	0.009*
Furosemid	0.083
Torasemid	<0.01*
Indapamid	0.188
Metolazon	0.645
Eplerenon	0.542
Diamox	0.450

In multivariate cox regression analysis use of diuretic therapy was not associated with mortality (Table 3a). This lack of an association was also observed for the different diuretic medicines (Table 3b). Age, ICU admission and creatinine were the only factors associated with mortality in this cohort (Table 3).

**Table 3:** Multivariate cox regression analysis for the association of different parameters with mortality, A) with diuretic therapy as pooled parameter and B) with the respective substances (*p < 0.05)

A		
	OR (CI)	*p*-value
Age	0.97 (0.96;9.97)	<0.01*
Black ethnicity	0.77 (0.19;3.15)	0.72
Creatinine	0.79 (0.74;0.85)	<0.01*
ICU admission	0.11 (0.10;0.15)	<0.01*
Diuretic therapy	1.11 (0.85;1.45)	0.43
B		
	OR (CI)	*p*-value
Age	0.97 (0.96;9.97)	<0.01*
Black ethnicity	0.77 (0.19;3.15)	0.72
Creatinine	0.79 (0.74;0.85)	<0.01*
ICU admission	0.11 (0.10;0.15)	<0.01*
Chlortalidon	0.52 (0.21;1.31)	0.16
Spironolactone	0.89 (0.52;1.53)	0.67
Torasemid	0.86 (0.62;1.20)	0.38
Furosemid	0.94 (0.48;1.83)	0.86

## Discussion

Over a 2-year period, more than 20,000 patients seen in our ER at a large tertiary care hospital for different reason. A total of 11 % of subjects received concomitant diuretic treatment, and 3 % were taking more than one diuretic agent. Diuretic therapy was not an independent risk factor for mortality in these patients.

Diuretic therapy has been investigated in multiple ways of either beneficially influencing heart failure, acute kidney injury or by potentially increasing mortality by increasing electrolyte disorders [10–12]. Our study demonstrates associations of diuretic agents as torasemide, spironolactone or chlortalidon with mortality in univariate analysis. However, this finding was not robust in a multivariate regression model. Therefore, one may raise the suspicion that the risk associated with diuretic therapy is rather related to the associated electrolyte disorders than to the substances itself [9]. Interestingly, furosemide itself was not associated with mortality. However, its dosage was. Our results are in line with those obtained in patients who underwent surgical procedures and consecutive acute kidney injury, where diuretic therapy was not associated with a higher mortality [6]. We relate these findings to the fact that by increasing renal insufficiency the dosage of furosemide increases and therefore mortality raises. We could not detect any difference between patients receiving diuretic therapy and those without in overall mortality and mortality with 28 days of hospital admission. This finding further strengthens the hypothesis that mortality in critical ill patients is rather related to electrolyte disorders or the higher morbidity of patients with existing diuretic therapy.

Age, ICU admission and creatinine were dominant risk factors in our study. These results are in line with other studies [6–8]. In these studies post-surgical patients with or without acute kidney injury were observed [6–8]. The results suggested a potential influence of diuretic therapy on mortality. However, these studies included markedly older patients and patients who underwent major surgical procedures [6–8]. Further, in almost all of the prior studies patients with acute kidney injury or the association with this condition and diuretic use was investigated [6–8]. We could not detect any association of the respective substances with mortality.

One limitation of our study is that it was not practicable to obtain data on and evaluate all medications the patients were taking because of the large number of patients included. Information on other substances that may influence serum electrolytes apart from diuretics, such as angiotensin-converting enzyme inhibitors, was not available. Further, differences regarding admission groups e.g. with specific cancer or coronary heart disease might be present. However, our classification of admission diagnoses only covered wide topics as cardiovascular or infectious diseases.

One of the strengths of the study, however, was the large number of patients included, and we therefore do not expect that these limitations had a substantial effect on our findings.

## Conclusion

In daily use, diuretic therapy appears a common first line therapy for many patients. Regarding electrolyte imbalances they should always be subscribed with caution. However, our results suggest that diuretic therapy is not an independent risk factor for mortality in patients admitted to an emergency department.

## Abbreviation

ICU: Intensive care unit
ER: Emergency room
SD: Standard deviation
CI: Confidence interval
ICD: International classification of diseases

## References

[1] Rose BD, Post TW. Clinical Physiology of Acid–base and Electrolyte Disorders. Volume 15 5. New York, NY: McGraw Hill; 2001. Clinical use of diuretics.

[2] Mann SJ (2008). The silent epidemic of thiazide-induced hyponatremia. J Clin Hypertens (Greenwich).

[3] Gross P, Palm C (2005). Thiazides: do they kill?. Nephrol Dial Transplant.

[4] Siegel D, Hulley SB, Black DM, Cheitlin MD, Sebastian A, Seeley DG (1992). Diuretics, serum and intracellular electrolyte levels, and ventricular arrhythmias in hypertensive men. JAMA.

[5] Leung AA, Wright A, Pazo V, Karson A, Bates DW (2011). Risk of thiazide-induced hyponatremia in patients with hypertension. Am J Med.

[6] Uchino S, Doig GS, Bellomo R, Morimatsu H, Morgera S (2004). Diuretics and mortality in acute renal failure. Crit Care Med.

[7] Wu VC, Lai CF, Shiao CC, Lin YF, Wu PC, Chao CT (2012). Effect of diuretic use on 30-day postdialysis mortality in critically ill patients receiving acute dialysis. PLoS One.

[8] Metz LI, LeBeau ME, Zlabek JA, Mathiason MA (2009). Acute renal failure in patients undergoing cardiothoracic surgery in a community hospital. WMJ.

[9] Arampatzis S, Funk GC, Leichtle AB, Fiedler GM, Schwarz C, Zimmermann H (2013). Impact of diuretic therapy-associated electrolyte disorders present on admission to the emergency department: a cross-sectional analysis. BMC Med.

[10] Wu MY, Chang NC, Su CL, Hsu YH, Chen TW, Lin YF (2014). Loop diuretic strategies in patients with acute decompensated heart failure: a meta-analysis of randomized controlled trials. J Crit Care.

[11] Danjuma MI, Mukherjee I, Makaronidis J, Osula S (2014). Converging indications of aldosterone antagonists (spironolactone and eplerenone): a narrativereview of safety profiles. Curr Hypertens Rep.

[12] Roush GC, Kaur R, Ernst ME (2014). Diuretics: a review and update. J Cardiovasc Pharmacol Ther.

